# Streptococcus equi subspecies zooepidemicus: an emergent cause of meningoencephalitis in Northeastern Brazil

**DOI:** 10.1590/S1678-9946202466051

**Published:** 2024-08-26

**Authors:** Luis Arthur Brasil Gadelha Farias, Marcos Maciel Sousa, Karene Ferreira Cavalcante, Marina Pinheiro Catunda Jucá, Aldenise de Olinda Castro, Liana Perdigão Mello, Rafael Ferreira Mesquita, Silviane Praciano Bandeira, Ana Paula Marchi, Tânia Mara Silva Coelho, Antônio Silva Lima, Érico Antonio Gomes de Arruda, Silvia Figueiredo Costa, Maura Salaroli de Oliveira, Lauro Vieira Perdigão

**Affiliations:** 1 Universidade de São Paulo, Hospital das Clínicas, Departamento de Doenças Infecciosas, Laboratório de Investigação Médica (LIM 49) São Paulo, São Paulo, Brazil; 2 Hospital São José de Doenças Infecciosas, Fortaleza, Ceará, Brazil; 3 Laboratório Central de Saúde Pública, Fortaleza, Ceará, Brazil; 4 Serviço de Verificação de Óbitos, Fortaleza, Ceará, Brazil; 5 Universidade Federal do Ceará, Programa de Pós-Graduação em Saúde Coletiva, Fortaleza, Ceará, Brazil; 6 Secretaria de Saúde do Estado do Ceará, Fortaleza, Ceará, Brazil

**Keywords:** Meningoencephalitis, Streptococcus equi, Streptococcus zooepidemicus, Bacteremia, Outbreak

## Abstract

This study describes an outbreak of *Streptococcus equi* subspecies *zooepidemicus* infections that caused meningoencephalitis and bacteremia related to unpasteurized milk consumption in northeastern Brazil. Epidemiological investigations and a brief literature review were conducted. Strains with possible neurotropism had not been identified in Brazil before these cases; however, in 2023, another case of meningoencephalitis caused by *Streptococcus equi* sp. *zooepidemicus* was described, revealing the need to maintain surveillance and highlighting that these neurotropic strains continue to circulate in the environment.

## INTRODUCTION

*Streptococcus equi* subspecies *zooepidemicu*s is a Gram-positive, β-hemolytic coccus, Lancefield group C streptococcal bacteria that is part of the commensal microbiota in horses and is an opportunistic pathogen in humans^1^. It represents one of three *S. equi* subspecies, along with *S. equi* sp. *equi* and *S. equi* sp. *ruminatorum*, which are equine pathogens^1^. *S. zooepidemicus* may infect domestic animals, such as dogs and cattle, economic animals, such as swine and goats, and wildlife. Human infections are usually related to expositional factors, such as contact with horses, consumption of contaminated unpasteurized milk and dairy products^2^.

Human infections are rare and may manifest as bacteremia, glomerulonephritis, pharyngotonsillitis, rheumatic fever, meningoencephalitis, and sepsis, which can evolve to death^2^. Most related publications are case reports or outbreaks with epidemiological correlations^3,4^. Outbreaks of *S. zooepidemicus* have been reported since the 1980s in countries such as Finland and Italy, most of which are highly positive in blood cultures and show a clear history of contact with horses and unpasteurized dairy products consumption^1,3,4^. Outbreaks of glomerulonephritis triggered by this pathogen have been described in Brazil; however, strains that cause other manifestations are rarely seen^3-5^. Most of these outbreaks occurred in Southeastern Brazil, and counterintuitively, meningoencephalitis cases are usually reported as isolated cases related to the consumption of unpasteurized milk or cheese. There are few outbreaks of meningoencephalitis caused by this pathogen identified via surveillance and molecular methods.

This study aimed to characterize the clinical presentation of five patients with *S. equi* infection and perform a brief literature review to convey its clinical relevance.

## MATERIALS AND METHODS

### Study design

This case series analyzed the medical records of patients with meningoencephalitis caused by *S. zooepidemicus* who were admitted to the Hospital Sao Jose de Doencas Infecciosas (HSJ) and a private clinic in Fortaleza, Ceara State, Brazil.

### Ethical statement

This study is part of a larger cohort was approved by the Research Ethics Committee of the Hospital Sao Jose de Doencas Infecciosas (HSJ) (protocol Nº CAAE 52811521.7.0000.5044).

### Data collection

Clinical and therapeutic data were systematically collected from the physical medical records and the hospital’s electronic record system, *ARS VITAE*, then analyzed by the researchers. Epidemiological data, clinical information, laboratory tests, and treatments performed at the center during the same period were analyzed.

### Laboratorial analysis

The laboratory investigation was initially conducted using the Vitek 2 compact platform (bioMérieux^®^), which employed individual identification cards that consisted of 64 wells with lyophilized biochemical tests. These tests allow for individual identification without the need for reagent addition. The obtained phenotype was 98% compatible with *Streptococcus equi* sp. *zooepidemicus*. However, a new identification process using proteomics, specifically matrix-assisted laser desorption/ionization time-of-flight mass spectrometry (MALDI-TOF MS), is required to further identify the isolate. Although MALDI-TOF MS is known for its high discriminatory power and superior mass/load ratio, the results obtained using this method are inconclusive. Thus, the VITEK MS PRIME (bioMérieux^®^) system was used for this purpose. The phenotype showed a split of 50% for *Streptococcus equi* sp. *zooepidemicus* and 50% for *S. equi* sp. *ruminatorum*, indicating close genetic proximity between these subspecies. The antibiotic susceptibility of Gram-positive pathogens was determined using a Vitek 2 card (AST-ST03), and the results were interpreted following the EUCAST rules^6^. Conventional biochemical and Lancefield group C antigen tests were not performed. FilmArray Meningitis Encephalitis Panel (bioMérieux, Marcy l’Étoile) was performed when available from the cerebrospinal fluid (CSF) samples.

## RESULTS

In July 2019, within a one-month period, an outbreak was identified in Ceara State, Northeastern Brazil. We found three patients with central nervous system infections that were characterized by meningoencephalitis and one patient with severe disseminated disease that was caused by *S. equ*i sp. *zooepidemicus*. In July 2023, a new case of a patient with the same clinical syndrome was identified, with the same pathogen causing meningoencephalitis (Table 1).

**Table 1:** The main clinical-epidemiological characteristics of the five patients reported herein.

Patient	Year	Age	Sex	Clinical Syndrome	Origin	Epidemiological Link	Outcome
1	2019	59	M	Meningoencephalitis	Horizonte municipality - Ceara State (rural area)	Artisanal cheese factory worker	Death
2	2019	32	F	Meningoencephalitis	Fortaleza municipality - Ceara State (urban area)	Consumption of cheese without sanitary control	Cure
3	2019	29	M	Meningoencephalitis and Infectious Endocarditis	Horizonte municipality - Ceara State (rural area)	Artisanal cheese factory worker and brother of Patient 1	Cure
4	2019	48	M	Pericarditis	Fortaleza municipality - Ceara State (urban area)	Unknown, but cousin of Patient 3	Cure
5	2023	49	M	Meningoencephalitis	Itapaje municipality - Ceara State (rural area)	Consumption of cheese without sanitary control	Cure

### Patient 1

A 59-year-old man, who worked in cheese production, at the Horizonte municipality, inland Ceara State, located 43 km from the capital Fortaleza in Northeastern Brazil, was admitted with fever, headache, and head stiffness. During physical examination, the patient was disoriented and drowsy; thus, CSF was collected, and the patient was referred to the intensive care unit due to sensory loss. The CSF analysis revealed a 350 cells/mm^3^ white blood cell count (71% neutrophils), 54 mg/dL glucose level, and 155 mg/dL protein level; additionally, the microscopy stains revealed Gram-positive cocci in chains. Ceftriaxone 2 g every 12h was administered with dexamethasone 0.15 mg/kg IV every 6h to treat the pneumococcal meningitis. After two days, the patient developed clinical worsening, and the antibiotic therapy was changed to cefepime 2 g every 8h and vancomycin 1 g every 12h. CSF culture revealed *S. equi* sp. *zooepidemicus* with susceptibility to ceftriaxone (minimum inhibitory concentration [MIC] 0.25 μg/mL), cefotaxime (MIC 0.25 μg/mL), vancomycin (MIC 0.5 μg/mL), linezolid (MIC ≤2 μg/mL), levofloxacin (MIC 1 μg/mL), and chloramphenicol (MIC 2 μg/mL). The FilmArray Meningitis Encephalitis Panel (bioMérieux, Marcy l’Étoile) evaluation of the CSF was negative for *Streptococcus pneumoniae, Streptococcus agalactiae, Neisseria meningitidis, Haemophilus influenzae, Listeria monocytogenes, Escherichia coli K1*, herpesvirus (1, 2, and 6), *Enterovirus*, Varicella-zoster virus, *Human parechovirus*, cytomegalovirus, and *Cryptococcus neoformans/gattii* complex. Blood cultures (two samples) were negative, and approximately one week later, the patient’s consciousness improved, although the fever and lethargy persisted. A systolic murmur was identified, and transthoracic echocardiography showed vegetation in the anterior leaflet of the mitral valve; thus, infective endocarditis (IE) was diagnosed. Unfortunately, the patient died before surgical treatment.

### Patient 2

A 32-year-old previously healthy female from Fortaleza, the capital city of Ceara State, was admitted with fever, head stiffness, drowsiness, and disorientation. Her physical examination revealed Brudzinski and Kernig signs and sixth cranial nerve involvement. During the epidemiological evaluation, the patient reported buying cheese sold on the street. CSF was collected, revealing 13.884 cells/mm^3^ white blood cell count (90% neutrophils), 56 mg/dL glucose level, and 45 mg/dL protein level; additionally, the microscopy stains were negative. Intravenous ceftriaxone 2 g every 12h and dexamethasone 0.15 mg/kg IV every 6h were administered. The patient showed clinical improvement during the following two days, until culture results were obtained. The CSF culture revealed *S. equi* sp. *zooepidemicus* with susceptibility to penicillin (MIC 0.06 μg/mL), ceftriaxone (MIC 0.25 μg/mL), and cefotaxime (MIC 0.25 μg/mL). FilmArray Meningitis Encephalitis Panel (bioMérieux, Marcy l’Étoile) evaluation of the CSF was also negative. The treatment was changed to penicillin G (4 million units every 4h for 10 days), and due to her similar history with that of Patient 1, a transthoracic echocardiogram was performed, and no signs of IE were found. The patient was discharged 20 days after the admission, with no sequelae found at the one-month follow-up.

### Patient 3

In the same month, a third patient presented, also from the same rural area in Northeastern Brazil as Patient 1. Notably, he was the brother of Patient 1 and worked in the same cheese factory. Approximately 15 days later, the patient presented to the emergency department with dyspnea, palpitations, dysarthria, and sensory loss. During the clinical investigation, the patient required intensive care unit (ICU) admission and broad-spectrum antibiotic therapy with piperacillin-tazobactam. Transthoracic echocardiography revealed congestive heart failure, and thoracic radiography revealed opacification compatible with pneumonia. The blood and urine samples tested negative, and the tracheal aspirate culture was also negative. A lumbar puncture was performed, and CSF analyses showed 11 cells/mm^3^ white blood cell count (95.1% lymphocytes), 85 mg/dL glucose levels, and 28.1 mg/dL protein levels, and the microscopy findings were negative. The CSF culture yielded *S. equi* sp. *zooepidemicus*, with a similar susceptibility profile. After clinical resolution, the patient was discharged.

### Patient 4

During the investigation in July 2019, active surveillance reported a fourth patient with a previous history of human immunodeficiency virus (HIV) infection and an epidemiological link with the one of the other (Patient’s 3 cousin) patients in Fortaleza municipality, Ceara State. The conditions at presentation were fever, thoracic pain, myalgia, and arthralgia. The electrocardiographic and transthoracic echocardiographic findings were consistent with pericarditis. *S. equi* sp. *zooepidemicus* strains were identified in the blood samples and pericardial effusion, and antiretroviral therapy (ART) was initiated with tenofovir/lamivudine/dolutegravir (TDF/3TC/DTG). Antibiotic therapy was administered using a combination of piperacillin-tazobactam 4.5 g every 6h and vancomycin (15 mg/kg/day). The pericarditis was treated conservatively, which lead to improvement.

### Patient 5

Approximately four years later, in 2023, a fifth case occurred, and the patient was registered on the regional surveillance system after four years. During this time, there were no other confirmed cases. Patient 5 was a 48-year-old male from another rural area, which was regionally close to the location of the previous patients and known as Itapaje city, Ceara State. Specifically, the region was located 129.3 km from Fortaleza city, and the patient was admitted with a fever, headache, and disorientation. On physical examination, the patient presented a Glasgow Coma Scale score of 8, and he had not protected his airways. The patient required immediate mechanical ventilation and ICU care, and the patient’s family reported that he sold dairy products and had contact with horses. After stabilization, due to the severity of his condition, ceftriaxone 2 g was administered every 12h with acyclovir 12.5 mg/kg per day, divided every 8 h, and dexamethasone 0.15 mg/kg, every 6h. CSF was collected and presented a 13.800 cells/mm^3^ white blood cell count (92% neutrophils) (Table 2), glucose level of zero, 868.50 mg/dL protein level, and 179 mg/dL lactate level; additionally, the microscopy stains presented Gram-positive cocci in chains. The FilmArray Meningitis Encephalitis Panel (bioMérieux, Marcy l’Étoile) evaluation of the CSF identified *Streptococcus pneumoniae*, whereas the CSF culture identified *S. equi* sp. *zooepidemicus*. MALDI-TOF-MS revealed a 50% split between *S. equi* spp. *zooepidemicus* and *ruminatorum*. The patient was susceptible to penicillin G, ceftriaxone, chloramphenicol, and vancomycin (MICs were not available) and was discharged after seven days of antibiotic therapy. During the six-month follow-up, the patient presented no sequelae.

**Table 2:** The cerebrospinal fluid analysis performed for the patients with meningoencephalitis reported herein.

Patient	Cell count (cells/mm^3^) (RV< 5)	Differential (%)	Proteins (mg/dL) (RV 15–45)	Glucose (mg/dL) (RV >2/3 serum glucose)	Gram Stain	Culture	Susceptibility Profile
1	350	71% neutrophils	155	54	Gram-positive cocci arranged in chains	*S. zooepidemicus*	Ceftriaxone (MIC 0.25μg/mL) Cefotaxime (MIC 0.25μg/mL) Vancomycin (MIC 0.5μg/mL) Linezolid (MIC ≤2 μg/mL) Levofloxacin (MIC 1 μg/mL) Chloramphenicol (MIC 2 μg/mL)
2	13,884	90% neutrophils	45	56	Negative	*S. zooepidemicus*	Penicillin (MIC 0.06μg/mL) Ceftriaxone (MIC 0.25μg/mL) Cefotaxime (MIC 0.25μg/mL)
3	11	95.1% lymphocytes	85	28.1	Negative	*S. zooepidemicus*	Penicillin (MIC 0.06μg/mL) Ceftriaxone (MIC 0.25μg/mL) Cefotaxime (MIC 0.25μg/mL)
5	13,800	92% neutrophils	868.5	0	Gram-positive cocci arranged in chains	*S. zooepidemicus*	Susceptible to Penicillin G, ceftriaxone, chloramphenicol, and vancomycin (MICs were not available*)*

RV = Reference values.

## DISCUSSION

*S. zooepidemicus* is an emerging opportunistic zoonotic pathogen associated with severe and life-threatening diseases and is a new cause of meningoencephalitis and bacteremia in humans. It causes diseases in humans via contact with infected animals and consumption of milk and dairy products. Outbreaks induced by emergent zoonotic pathogens represent a health risk for many individuals if quality controls in milk production are not followed and mainly occurs in rural areas, such as Northeastern Brazil. In Brazil, most outbreaks are related to milk-derived products, with well-described manifestations such as triggering glomerulonephritis^3-5^. The first large outbreak related to this pathogen occurred in 1998 in Nova Serrana municipality, Minas Gerais State, in Southeastern Brazil^4^. Of the 134 patients with *S. zooepidemicus*, four died, all of whom had contact with the same unpasteurized milk products^4^. Other municipalities in Minas Gerais State, such as Guaranesia and Monte Santo municipalities, have also experienced glomerulonephritis outbreaks^3-5^. More recently, in 2018, the same strains previously found in Nova Serrana municipality were identified and studied during the Monte Santo outbreak. These strains were identified as *S. zooepidemicus* SzPHV5 and were likely to show superior nephritogenic properties compared to other superficial zone protein (SZP) types^4,5^. Notably, information from the outbreaks in the northeastern region were not available until this report. Table 3 summarizes the outbreaks and cases recorded in Brazil due to *S. zooepidemicus*.

**Table 3:** Summary of outbreaks and cases reported in Brazil due to *S. zooepidemicus*.

Article	Study type	Study year	Place	Year of Outbreak/Case	Number of affected individuals	Clinical Syndrome	Diagnostic Methods	Strain
Sesso *et al.* ^4^	Case-control	2005	Nova Serrana municipality - Minas Gerais State	1998	135	Glomerulonephritis	Cultures of oropharyngeal swabs	N/A
Torres *et al.* ^5^	Retrospective study	2018	Monte Santo municipality - Minas Gerais State	2012	175	Glomerulonephritis	Cultures of oropharyngeal swabs, PCR, and sequencing analysis	SzPHV5
This article	Case series	2024	Fortaleza municipality - Ceara State	2019 and 2023	5	Meningoencephalitis; Pericarditis, and Infectious endocarditis	CSF and blood Cultures	N/A

N/A = Not available.

Other outbreaks in Brazil have been related to dairy products, such as cheese and other contaminated unpasteurized milk products. In Brazil, the artisanal production of cheese and other dairy products is an important part of the cultural scope of certain regions, such as Northeastern Brazil. Aiming at safety and good agricultural practices, the Brazilian Federal Law 13,860 in June 2019 stated that artisanal cheese makers are responsible for the health and safety of the cheese they produce^7^. This law restricts the production of artisanal cheese from raw milk (*in natura*) to cheese factories located in rural establishments that are certified as free of tuberculosis and brucellosis. Unfortunately, many areas still produce dairy products without certification, mainly due to a lack of sanitary inspection. Notably, to produce artisanal cheese, the establishment must hold a registration issued by the inspection body of the state or municipality where the enterprise is duly installed^7^.

On the other hand, although we present four cases of meningoencephalitis due to this pathogen that occurred over a five-year period in a region with no previously reported cases, this manifestation is scarcely described in the literature. Eyre *et al*.^8^ published a review of 20 cases of patients with meningitis due to *S. zooepidemicus* that had been reported worldwide, up until 2010. Most cases occurred in the USA and Europe, being mainly related to contact with horses or contaminated milk. Subsequently, a few other cases were reported in 2011 and 2013 in the USA and Peru, respectively^9,10^. Notably, none of these cases were from Brazil or related to a specific strain.

Cases of meningitis have also been reported in other Latin American countries such as Cuba, Costa Rica, and Uruguay^11-13^. In 2015, Cuba recorded its first case, with a 50-year-old male patient with meningitis due to *S. zooepidemicus* with a fatal outcome^11^. More recently, a five-month-old Costa Rican patient developed a clinical presentation of meningoencephalitis and sepsis due to this pathogen. After further investigation, it was confirmed as the first infection by *S. zooepidemicus* in Costa Rica^12^. In Uruguay, cases of pneumonia, meningitis, and sepsis caused by this pathogen have been described^13^. The characterization of the strains revealed isolates containing the lnuB gene responsible for the L phenotype, which is different from the strains found in Brazil^4,5,13^. To the best of our knowledge, this is the first description of *S. zooepidemicus* causing a neurological syndrome in Brazil, resembling the emergence in other Latin American countries.

During the Brazilian outbreak in July 2019, one of four patients died of bacteremia and sepsis. The mortality rate associated with meningitis secondary to *S. zooepidemicus* infection is 24% and, according to Eyre *et al*.^8^, only 38% of survivors achieve complete recovery. The new case reported in 2023 reinforces the need to maintain surveillance and understand that these neurotropic strains are continuing to circulate in the environment.

Understanding the profile of the patients reported here in light of their health conditions is especially important. Other *streptococci* strains, such as *S. suis*, which relate to pig breeding, slaughtering, and consumption, have also been reported in rural areas^14,15^. For example, another similar study reported a case of *S. suis* in a pig farmer from Bahia with health implications related to the complex interactions between the environment, humans, and domestic and wild animals in Northeastern Brazil that need to be better studied. With the increase in diagnostic methods and microbiological technologies, newly emerging pathogens are being identified more easily each year^16^. The implications and origins of these pathogens must be discussed and investigated. CNS infections of zoonotic origin have increased, and understanding the spatiotemporal distribution of pathogens in wildlife is key to identifying the dynamics and predicting the establishment of these infections^16^.

Our study shows several limitations that should be noted when interpreting our findings. First, this is a small case series with a small sample size, despite being a representative and unique sample of patients in Northeastern Brazil who were not previously reported. It should be noted that this study does not provide a comprehensive outbreak report, and other patients with mild or different manifestations without proper documentation may have occurred during the outbreak period. Another limitation was the absence of whole-genome sequencing data, which would improve our understanding of the similarities and differences between strains from different regions of Brazil and worldwide. The samples collected from milk and cheese from Horizonte municipality, Ceara State, yielded negative results for *S. zooepidemicus*, although the samples were collected months after disease onset.

## CONCLUSION

This study presents the emergence of a zoonotic pathogen in Northeastern Brazil with infrequent neurological repercussions and apparent neurotropism. This outbreak and the emergence of the pathogen as a cause of clinical meningoencephalitis highlight the importance of being aware of this pathogen and the risks related to unpasteurized milk consumption. Surveillance using molecular methods, when available, would be useful to better understand the characteristics, pathogenicity, and risks associated with these strains. The strains isolated in this study appeared to be more neurotropic than those previously described from Southeastern Brazil. To the best of our knowledge, this is the first outbreak of meningoencephalitis caused by *S. zooepidemicus* in Northeastern Brazil and likely provides the first evidence of a neurotropic strain in the region.
